# The Role of Serum 5-HIAA as a Predictor of Progression and an Alternative to 24-h Urine 5-HIAA in Well-Differentiated Neuroendocrine Neoplasms

**DOI:** 10.3390/biology10020076

**Published:** 2021-01-21

**Authors:** Maria Wedin, Sagar Mehta, Jenny Angerås-Kraftling, Göran Wallin, Kosmas Daskalakis

**Affiliations:** Department of Surgery, Faculty of Medicine and Health, Örebro University, 701-85 Örebro, Sweden; maria.wedin@regionorebrolan.se (M.W.); sagar.mehta@regionorebrolan.se (S.M.); jenny.angeras-kraftling@regionorebrolan.se (J.A.-K.); goran.wallin@regionorebrolan.se (G.W.)

**Keywords:** neuroendocrine neoplasm, biomarkers, 5-HIAA

## Abstract

**Simple Summary:**

5-hydroxyinoloacetic acid (5HIAA) is the breakdown product of serotonin and it is traditionally measured in 24-h urinary samples in patients with neuroendocrine neoplasms (NENs). 5HIAA measurement in patient serum has recently become available and has started replacing the traditional method in many centers, as it is more convenient and often preferred by patients. In this prospective, single center study, we aimed to investigate the clinical utility of serum 5HIAA for diagnostic purposes and disease surveillance in a cohort of patients with well-differentiated NENs. Our analysis confirmed an association between serum 5HIAA and the presence of liver metastases, as well as the extent of liver tumor involvement, demonstrating that the biomarker becomes positive in advanced disease stages. However, there was no evident association between a change in serum 5HIAA and change in disease status. Additionally, with respect to diagnostic purposes as compared to urinary 5HIAA testing, there was a substantial agreement between the two methods. In conclusion, serum 5HIAA performs well compared to urinary testing for diagnostic purposes but does not seem adequate as a solo biomarker of disease progression.

**Abstract:**

Our aim was to investigate the clinical utility of serum 5HIAA for disease surveillance and diagnostic purposes in a cohort of patients with well-differentiated neuroendocrine neoplasms (WD-NENs). Forty-eight patients with WD-NENs and concurrent serum and urinary 5HIAA testing, as well as CT/MRI imaging, were included. Analysis of matching-pairs did not reveal any association between RECIST 1.1 responses and changes in serum 5HIAA levels (*p* = 0.673). In addition, no correlation was evident between RECIST 1.1 responses and >10%, >25% or >50% changes in serum 5HIAA levels (Fisher’s exact test *p* = 0.380, *p* > 0.999, and *p* > 0.999, respectively). The presence of liver metastases and extensive liver tumor involvement were associated with higher serum 5HIAA levels (*p* = 0.045 and *p* = 0.041, respectively). We also confirmed a strong linear correlation between the measurements of serum and urine 5HIAA (*n* = 24, r = 0.791, *p* < 0.0001). The concordance rate of serum and urinary 5HIAA positivity at standardized laboratory cut-offs was 75%. In patients with normal renal function tests, the concordance between the two methods was as high as 89%, and a sensitivity and specificity of 80% and 88.9%, respectively, was evident (Cohen’s kappa coefficient = 0.685). In conclusion, serum 5HIAA performs well compared to urinary testing for diagnostic purposes, mainly in advanced disease stages, and corresponds well to liver tumor burden. However, it is not adequate to predict tumor progression.

## 1. Introduction

Neuroendocrine neoplasms (NENs) comprise a heterogeneous group of tumors with a rising incidence in recent years [1]. Although the majority of patients with well-differentiated NENs (WD-NENs) present with metastatic disease, many undergo surgery and/or multimodal treatments with a generally favorable prognosis [2,3]. Nevertheless, WD-NEN patients undergo long surveillance to detect disease recurrence/progression and monitor treatment response with a great variation in the type (biochemical testing, cross-sectional/functional imaging) and intensity of the applied follow-up strategy [4].

Functioning WD-NENs may secrete a wide range of hormones, amines, and peptides. Elevated concentrations of these products can cause relevant secretory syndromes, but can also be used as biomarkers for diagnosis and subsequent patient surveillance. The carcinoid syndrome (CS) becomes manifest when vasoactive substances from the tumors, such as serotonin, tachykinins, bradykinins, kallikrein, histamine, vasoactive intestinal peptide (VIP), prostaglandins, and substance P enter the systemic circulation escaping hepatic degradation. CS is encountered mainly in patients with small intestinal NENs (SI-NENs), but also in patients with lung NENs (LNENs) and less commonly with pancreatic NENs (PanNENs). Notably, serotonin and other active peptides are metabolized during first-pass effects in the liver. Therefore, CS generally only manifests once liver or other systemic metastases are present. Patients with CS may suffer from a constellation of symptoms including cutaneous flushing, gastrointestinal hypermotility with diarrhea, carcinoid heart disease, and bronchial constriction [5]. In the setting of PanNEN, patients may exhibit positive immunostaining for hormones, neuropeptides, and amines, including serotonin (5-HT) in 4–8% of the cases, albeit not always functioning, which is clinically associated with CS [6,7].

The currently used general markers for NEN patients in clinical practice are chromogranin A (CgA) and 5-hydroxyindoleacetic acid (5HIAA). Although CgA is a sensitive marker for NENs and correlates well to tumor burden and survival, it is rather unspecific since it is co-secreted with peptide hormones from neuroendocrine cells of several organs [8,9,10]. In addition, it may be elevated in multiple other conditions, such as renal failure, cardiac and inflammatory diseases, and proton pump inhibitor medication [11]. Although plasma CgA and urinary 5HIAA have traditionally been used in disease diagnosis and surveillance, a recent study has demonstrated a weak association between plasma CgA concentrations and changes in disease status, challenging the role of plasma CgA for treatment monitoring [10]. On the other hand, 5HIAA is the breakdown product of 5-HT and is traditionally measured in 24-h urinary samples. Urinary 5HIAA analysis, apart from being time consuming and more inconvenient for the patients, is also quite prone to sampling errors [12]. Importantly, urinary 5HIAA is a highly specific marker, albeit with lower sensitivity compared to CgA, as it commonly becomes elevated at late disease stages, when metastases have already occurred [9,11]. Serum 5HIAA measurement has recently become available and has started replacing the traditional method of urine collection in many centers, as it is often preferred by patients. Nevertheless, several laboratories have faced safety and reproducibility issues, as urinary samples should be collected into acid-containing bottles with incomplete sampling, often giving misleading results [13]. In addition, as 5HIAA is eliminated in the urine, patients with renal insufficiency may have false negative results following urinary 5HIAA sampling.

To our knowledge, there are no studies on the prognostic significance of serum 5HIAA levels, and only few studies exist that confirm the diagnostic value of this marker, as compared to the 24-h urinary sampling method [13,14,15]. The primary endpoint of the present study was to compare the association between changes in serum 5HIAA levels and the findings of computed tomography/magnetic resonance imaging (CT/MRI) scans, assessing specifically whether serum 5HIAA accurately depicts changes in disease status (regression/stable disease or progression). The study’s secondary endpoints were to determine the association of serum 5HIAA with patient- and tumor-related parameters, including disease stage and tumor burden; to further validate the clinical utility of measuring 5HIAA in patient serum by assessing the correlation and the diagnostic concordance between paired urinary and serum 5HIAA test results; and finally, to investigate the impact of impaired renal function in serum 5HIAA measurements.

## 2. Materials and Methods

In this prospective, observational, non-randomized study, we consecutively included 48 NEN patients followed at a single tertiary referral center (Department of Surgery, Örebro University Hospital, Sweden) from June 2017 to July 2020. We included only patients with confirmed histopathology of WD-NEN of small intestinal, lung, pancreatic, or of unknown origin and available urinary and serum 5-HIAA measurements.

### 2.1. Ethics Statement

All procedures performed in studies involving human participants were in accordance with the ethical standards of the institutional and/or national research committee and with the 1964 Helsinki declaration and its later amendments or comparable ethical standards. Swedish Ethical Review Authority approval was obtained (DNR 2020-00539). Written informed consent was obtained from the study participants.

### 2.2. Disease Classification

Tumor grade was determined from primary and lymph node specimens and/or liver biopsies according to the Ki-67 proliferation index. We used the 2015 and 2017 WHO classification systems, respectively, for grading lung and gastro-enteropancreatic NENs [16,17]. We applied the Charlson comorbidity index to assess patients’ comorbidities [18]. For staging, we used the 8th edition of the American Joint Committee on Cancer (AJCC) [19]. Patients’ medications were monitored by dedicated specialists. To ensure the quality of data reporting, we followed the STROBE statement [20].

### 2.3. 5-HIAA and CT/MRI-Surveillance Protocol

The included NEN patients were subjected to baseline biochemical work-up as well as cross-sectional and functional imaging with somatostatin receptor scintigraphy or 68- Gallium-PET/CT at diagnosis, whereas those already followed, i.e., patients with residual or recurrent disease post-surgery and those with distant-stage disease were monitored with CT or MRI at varying time intervals, as per ENETS guidelines and clinically indicated per patient [21,22]. During these visits, follow-up laboratory tests with concurrent sampling of urine and serum 5-HIAA were obtained prospectively along with kidney function tests. Renal function tests were considered abnormal, when plasma-Creatinine concentration was >100 μmol/L and/or eGFR was <50 mL/min/1.73 m^2^. Serum 5HIAA was measured within 3 months of a CT/MRI in patients undergoing at least 2 sequential examinations within a period of 1–24 months. Patients were instructed to avoid foods rich in serotonin that would interfere with 5-HIAA measurement 3 days prior to and during sample collection. Drugs that could potentially affect metabolism of serotonin and thus the 5-HIAA test were also discontinued. CT/MRI followed standardized NEN examination protocols [23]. Morphological imaging of the abdomen (CT or MRI) was further reviewed, and the highest liver tumor load (LTL) was recorded. The following staging system was used to describe the stage of liver involvement: stage 1 =< 5 metastases confined in 1 lobe, stage 2 = bilobar and/or 5–10 metastases, and stage 3 => 10 metastases or diffuse metastatic disease.

### 2.4. Comparison Analysis of 5HIAA and CT/MRI

“Matching pairs” of s-5HIAA and CT/MRI assessments were defined for each patient to determine the biomarker’s ability to depict disease progression. In particular, biochemical changes in serum levels of 5HIAA were analyzed with respect to “matching changes” in tumor size, as defined by the RECIST1.1 for regression, stable disease, or progression [24]. The radiological assessment was made by a blinded radiologist with respect to the findings of the biochemical serum and/or urinary 5HIAA analysis. For urinary 5-HIAA levels, the reference value of 50μmol/L/24 h was used, whereas for serum 5HIAA we used the reference value of 123 nmol/L. A 25% change in serum concentration was set to distinguish between increased, unchanged, or decreased serum 5HIAA levels. The changes in serum 5HIAA levels were subsequently compared with the changes in tumor size on the corresponding CT/MRI (“matching pairs” of Δs5HIAA and 2 sequential CT/MRI scans). The same imaging technique was used in the individual patients to determine disease status. The urinary and serum 5HIAA samples were collected at varying intervals depending on differences in individual follow-up, as well as patient and/or responsible physician preferences.

### 2.5. Laboratory Specifications for Serum and 24-h Urine 5HIAA Measurement

With respect to serum 5HIAA analysis laboratory specifications, blood was collected in serum vacutainer tubes (BD Vacutainer; BD AB, Stockholm, Sweden) that were allowed to clot for at least 30 min, after which they were centrifuged for 7 min at 2000× *g* using a Hettich centrifuge (HETTICH Instruments LP, Beverly, MS, USA). Using a Tecan Evo 150 sampling robot (Tecan Group Ltd. Männedorf, Switzerland) 100 µL of serum supernatant was mixed with 400 µL methanol containing 100 ng/mL 5-HIAA-4,6,7-D3-3-acetic-D2 acid internal standard (Cerilliant, Round Rock, TX, USA) in a 96 well plate. The mixture underwent vortex mixing for 5 min. After 2 h of incubation at 8 °C the samples were centrifuged for 10 min at 4000× *g*. Samples were analyzed using a Waters Acquity UPLC system (Waters Chromatography Europe BV, Etten-Leur, The Netherlands) equipped with Waters HSS T3 column (1.8 µm, 2.1 × 100 mm) and a Waters Xevo TQ-S micro triple quadrupole MS-detector. Mobile phases A and B consisted of 0.1 % formic acid in water and 0.3 % formic acid in methanol, respectively. Quantification was performed after internal standard normalization against a standard curve of 5-Hydroxyindole-3-acetic acid (Sigma-Aldric, Saint Louis, MO, USA) at four levels (three plus blank). The method was linear at a range of 5–2000 nmol/L, with an extended linearity up to 10000 nmol/L. The mass spectrometric detection was performed using multiple reaction monitoring (MRM) with an electrospray ionization (ESI) source in positive mode. Results were calculated using the Waters MassLynx software. During method validation, a coefficient of variation (CV) for the total precision was measured at 9.0% at 45 nmol/L and 3.3% at 130 nmol/L.

Accordingly, with regards to urinary 5HIAA analysis, after 24-h urine collection, samples were acidified using 6 mol/L HCl and frozen at −80 Celsius Degrees. Before analysis, samples, controls and calibrators were thawed and centrifuged at 1500× *g* for 5 min using a Hettich Rotanta centrifuge (HETTICH Instruments LP, Beverly, Massachusetts, USA). Subsequently, internal standard, 5-hydroxy-2-indolecarboxylic acid (Sigma-Aldric, Saint Louis, MO, USA) was added to each sample. Samples were analyzed using a Waters HPLC Alliance 2695 (Waters Chromatography Europe BV, Etten-Leur, The Netherlands) with electrochemical detection. Results were calculated against a one-point standard curve with a 5-hydroxyindoleacetic acid calibrator from Bio-Rad (Bio-Rad, Hercules, CA, USA) using the Waters Empower software. The method was linear between 2–600 µmol/L. Method precision was measured as a coefficient of variation (CV) of 8.0% at 15 µmol/L and 6.0% at 130 µmol/L.

### 2.6. Statistics

The statistics software used throughout was GraphPad Prism, version 9 for Windows (GraphPad Software). Single-center institutional data were pooled and summarized. Exploratory analyses were performed using descriptive statistics. Non-parametric tests were applied due to distributions deviating from normal. Data are presented as medians with range, as appropriate for the number of samples indicated. The Mann-Whitney U test and the Fisher’s exact test in contingency table analyses were used to test the correlation between Δs5HIAA and tumor size regression/stable disease, and progression, respectively. A *p*-value less than 0.05 was considered statistically significant and 95% Confidence Intervals (CI) were provided in relevant analyses. The concordance of urine and serum 5HIAA measurements was presented. Inter-rater reliability and sensitivity/specificity for serum compared to urine collection for clinically established cut-offs were evaluated with Cohen’s Kappa coefficients. Bonferroni correction for multiple testing were conducted as appropriate.

## 3. Results

### 3.1. Baseline Characteristics

We included 48 consecutive patients diagnosed with WD-NEN (37 small intestinal NENs [SI-NENs]; 8 pancreatic NENs [PanNENs]; 1 lung NEN [LNEN]; and 2 of unknown primary origin [UPO]). Twenty-one of the patients displayed TNM stage IV. Twenty three patients had grade 1 and 19 patients grade 2 tumors (in six patients, the ki67 proliferation index of the tumor was not determined). A summary of the patient characteristics at baseline is presented in Table 1.

### 3.2. Changes in Serum 5HIAA Levels and Disease Status

**Figure 1:**
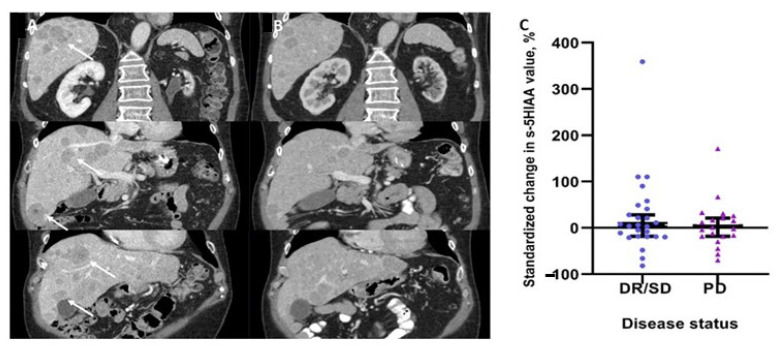
(**A**) Patient with small intestinal neuroendocrine neoplasm (SI-NEN). Liver metastases on coronary plane with evident progression (white arrows) according to RECIST 1.1, as compared to (**B**): CT examination of the same patient performed 5 months earlier. (**C**) Scatter plot of the standardized change in serum 5-hydroxyinoloacetic acid (5HIAA) (median with 95% CIs) in relation to disease progression (*p* = 0.673). Serum 5HIAA values were obtained concurrently to matched CT/MRI examinations to access disease status according to RECIST 1.1. Abbreviations. DR: Disease Regression; PD: Progressive Disease; RECIST: Response evaluation criteria in solid tumors; SD: Stable Disease.

Analysis of 48 matching pairs with respect to biochemical standardized changes in serum 5HIAA levels and paired CT/MRI assessments, did not reveal any significant change in serum 5HIAA values between patients with and without progressive disease (*p* = 0.673, Figure 1C). In addition, no correlation was evident between RECIST 1.1 responses and >10%, >25% or >50% changes in serum 5HIAA levels (Fisher’s exact test *p* = 0.380, *p* > 0.999 and *p* > 0.999, respectively). In the 21 patients with tumor progression according to RECIST1.1, the diagnostic sensitivity and specificity of an increased serum 5HIAA concentration were 40% and 53%, respectively. The positive and negative predictive values were 19 and 76%, respectively.

### 3.3. Serum 5HIAA Levels in Relation to Patient- and Tumor-Related Parameters

**Figure 2:**
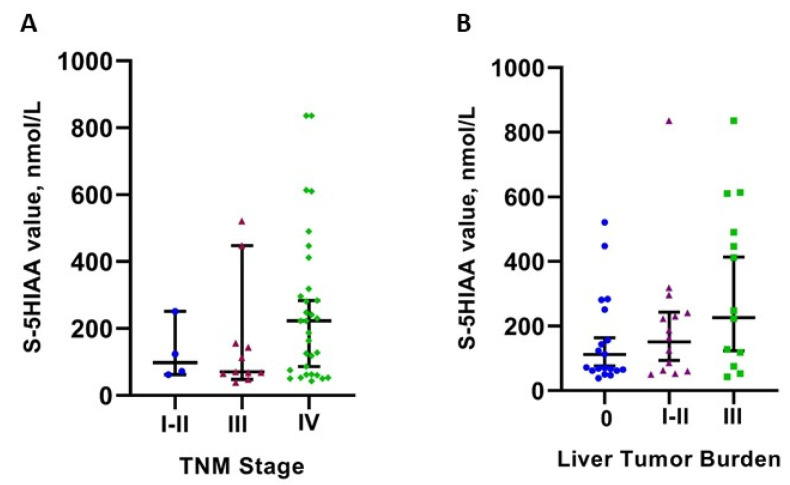
(**A**) Scatter plot of serum 5HIAA values (median with 95% CIs) in patients with different TNM stages (*p* = 0.398). Only four patients in the study cohort exhibited TNM stages I and II at the time of serum 5HIAA sampling. (**A**) Scatter plot of serum 5HIAA values (median with 95% CIs) in patients with different types of liver involvement (*p* = 0.041). Only two patients exhibited bilobar and/or 5–10 liver metastases at the time of serum 5HIAA measurement; hence types I and II in (**B**) were grouped together. Two outliers are not presented in (**A**,**B**) because their data points were outside the range of the *y* axis. The excluded values were: 3199 and 2076 nmol/L; both cases had TNM stage IV disease and extensive (type III) liver involvement.

**Figure 3:**
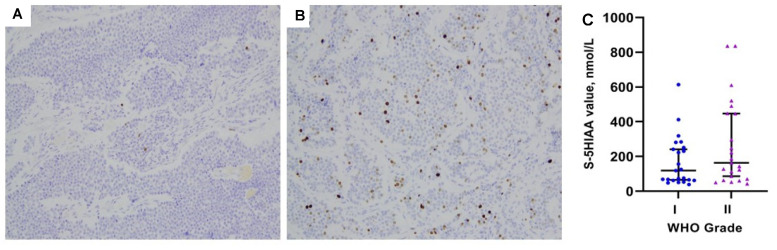
(**A**) Low Ki67 staining of a primary tumor in the distal ileum from a patient with a low-proliferative small intestinal neuroendocrine neoplasm (SI-NEN) in the study cohort. (**B**) High Ki67 staining from another patient with SI-NEN in our study cohort, also located in the distal ileum. (**C**) Scatter plot of serum 5HIAA values (median with 95% CIs) in patients with grade I (Ki67 < 3%) vs. grade II tumors (Ki67 3–20%; *p* = 0.174). Two outliers are not presented because their data points were outside the range of the y axis. The excluded values were: 3199 and 2076 nmol/L; both cases had grade II disease.

In the primary analysis of 48 ”matching pairs” of serum 5HIAA and CT/MRI assessments, there was a positive correlation between disease TNM stage and serum 5HIAA positivity at clinically used cut-offs of 123 nmol/L (Pearson Chi-square *p* = 0.017). However, this correlation with TNM stage was not confirmed in the Mann-Whitney U test analysis of serum 5HIAA values (*p* = 0.398; Table 2, Figure 2A). The presence and extent of liver metastases (liver tumor burden) though were linked with elevated serum 5HIAA values (Mann-Whitney U test: *p* = 0.045 and *p* = 0.041, respectively; Figure 2B). Further analysis of disease WHO grade in relation to serum 5HIAA levels did not reveal any significant association (*p* = 0.174; Table 2, Figure 3). Table 2 summarizes individual data analysis of patient- and tumor-related parameters in relation to serum 5HIAA values.

### 3.4. Correlation and Concordance of Urinary and Serum 5HIAA Values

**Figure 4:**
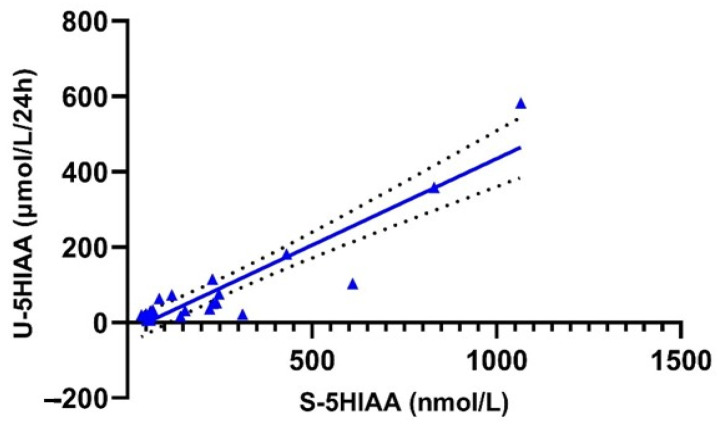
XY plot of urinary and serum 5HIAA values demonstrating a strong linear correlation (Spearman’s correlation test, *r* = 0.792, *p* < 0.0001).

Spearman´s correlation analysis demonstrated a strong linear correlation between serum and urinary 5-HIAA values of the included samples (*n* = 24, *r* = 0.791, *p* < 0.0001; Figure 4). The concordance rate of serum and urinary 5HIAA positivity at standardized laboratory cut-offs was 75% (19/24 cases). Serum 5HIAA sensitivity and specificity compared to that of urinary 5HIAA were 83.3% and 66.7%, respectively (Cohen’s kappa coefficient = 0.500). In patients without any impairment of the renal function (19/24), the concordance between the two tests was as high as 89% (17/19 cases). Serum 5HIAA sensitivity and specificity compared to that of urinary 5HIAA were 80 and 88.9%, respectively (Cohen’s kappa coefficient = 0.685). In cases with abnormal renal function tests, disconcordance between serum and urine 5HIAA positivity was as high as 60% (3/5 cases).

## 4. Discussion

In this prospective, single center, observational study, the role of serum 5HIAA for disease surveillance, therapy monitoring, and diagnostic purposes was evaluated in a cohort of patients with well-differentiated NENs. Specifically, we assessed if serum 5HIAA levels could be related to patient- and tumor-related parameters, if serum 5HIAA changes correpond to a transition from stable disease to progressive disease, and also investigated the diagnostic value of serum 5HIAA compared to the traditional method of urinary testing, paying special consideration to concurrent renal function tests. As the incidence of WD-NENs is rising and multiple treatment options have become available, there is a great need to identify suitable non-invasive markers for diagnostics but also to monitor treatment response. We demonstrated a significant association between elevated serum 5HIAA concentrations and the presence of liver metastases, as well as the extent of liver tumor involvement. On the other hand, there was no evident association between a change in serum 5HIAA levels and progression (RECIST 1.1). Finally, as compared to urinary 5HIAA testing, there was a substantial agreement and a strong linear correlation between the measurements of the two methods.

The prognostic value of urinary 5-HIAA has previously been assessed and its doubling time has been demonstrated as a potential risk stratification tool, possibly identifying patients with a high risk for disease progression; however, further validation in an independent cohort would be necessary [25]. In another study, urinary 5-HIAA levels as high as >10 times the upper limit of normal was an independent factor for survival [26]. Our data demonstrate the lack of predictive value of recently utilized serum 5HIAA in respect to disease progression. In particular, we were not able to demonstrate that changes in serum 5HIAA levels at different biomarker cut-offs predict RECIST 1.1 changes in disease status, raising concerns regarding the usefulness of serum 5HIAA in patient surveillance and monitoring treatment response. Indeed, serum 5HIAA measurement has very low sensitivity for progressive disease. Therefore, our findings did not support the use of 5HIAA as a clinically useful single marker of disease progression. However, serum 5-HIAA correlates with the presence of liver metastases, radiographic pattern, and extent of liver tumor burden. Therefore, it could be useful during the initial disease staging.

Our results suggest that serum 5HIAA could replace the traditional method of 24-h urinary sampling and testing for diagnostic purposes as it exhibits a strong linear correlation and substantial agreement with the latter. This is indeed in accordance with the findings of two recent studies on the diagnostic utility of serum 5HIAA in NENs [13,14,15]. Furthermore, blood-based markers are easily repeatable, whereas urinary 5-HIAA may be difficult to measure accurately in practice. In 5 patients with abnormal renal function tests, defined as a plasma-Creatinine concentration >100 μmol/L and/or eGFR < 50 mL/min/1.73 m^2^, serum and urine 5-HIAA were concordant in only 2 out of 5 cases. Hence, larger sample size would be required to validate whether serum and urinary 5-HIAA concentrations are disconcordant in patients with impaired renal function. On the other hand, the disconcordance observed in the two cases with normal renal function might be due to the fact that we could not perform serum and urinary sampling at the same day allowing for a short period of time (3–4 weeks) between the two tests.

Although serum 5HIAA has certain prognostic value as it becomes positive in advanced stages when liver metastases are present and depicts well liver tumor burden, it probably needs to be assessed in conjunction with CgA and other novel biomarkers as well as imaging and clinical parameters if it is to assist in the patients’ surveillance in the long-term at all. Nevertheless, in the subset of NEN patients that received systemic treatments, such as interferon-a, chemotherapy, molecular targeted therapies, as well as peptide receptor radionuclide therapy, higher s-5HIAA levels were present possibly owing to more advanced disease stages necessitating these treatment modalities. Thus, it is crucial for clinicians involved in the management of WD-NEN patients subjected to disease surveillance to apply follow-up strategies incorporating mainly cross-sectional and/or functional imaging modalities.

Our study has several limitations, the most important limitation being its design, which, because of selection bias and differences in clinicians’ preferences, may have excluded patients with more advanced disease. Moreover, NEN heterogeneity plus the inclusion of NENs originating both from the pancreas and the small intestine, as well as that of NENs of thoracic and unknown origin, may have confounded the results. Another limitation is the relatively small sample size of our study and the inclusion of WD-NEN patients at different time points in the disease course, when concurrent biochemical testing and imaging was available. Furthermore, concurrent urinary and serum sampling was available in half of the patients in the present study. Differences in prior surgical management and medical therapies in a subset of this cohort may also have confounded the results. In addition, partly due to insufficient length of follow-up, we did not explore the prognostic value of serum 5HIAA on progression-free or overall survival. Finally, the scarcity of NEN patients with renal impairement in the study cohort precludes any safe conclusions to be derived with respect to the impact of impaired renal function in 5HIAA testing. Importantly, the use of 5HIAA assays in patient serum and urine at our institution has not changed during the study period, being, therefore, one of the strengths of the present study. Nevertheless, blinded central assessment of responses according to 1.1 RECIST criteria was available at our institution.

## 5. Conclusions

In this prospective, single center study of WD-NEN patients, there was a significant association between serum 5HIAA and the presence of liver metastases, as well as the extent of liver tumor burden, confirming that the biomarker becomes positive in advanced disease stages. However, there was no evident association between a change in serum 5HIAA and change in disease status. Additionally, with respect to diagnostic purposes as compared to urinary 5HIAA testing, there was a substantial agreement and a strong linear correlation between the two methods. As serum 5HIAA performs well compared to urinary testing and accurately depicts tumor burden, it is well suited for patient diagnostics, mainly in patients with metastatic disease. However, it does not seem adequate to predict tumor progression. With respect to the impact of renal function in 5HIAA measurement, studies with larger sample size are warranted to elucidate whether impaired renal function affects serum 5-HIAA testing. In conclusion, with regards to patient surveillance and treatment monitoring, cross-sectional and/or functional imaging remains the preferred approach until further larger scale prospective, observational studies on currently available and/or novel biomarkers in the field of NENs become available.

## Figures and Tables

**Table 1:** Baseline patient and tumor characteristics of the study cohort.

Characteristics	*N* = 48	%
Gender		
Male	23	47.9
Female	25	52.1
Age, years (median, range)	71 (45–89)	-
Time since Initial Diagnosis, Months (Median, Range)	36 (0–288)	-
Inheritance		
Sporadic	42	87.5
Familial	6	12.5
Primary Tumor Site		
Small Intestine	37	77.1
Pancreas	8	16.7
Lung	1	2.1
UPO	2	4.2
Secretory Status		
Functioning	20	41.7
Non-Functioning	28	58.3
Grading		
G1 (Ki67 < 3%)	23	47.9
G2 (Ki67 3–20%)	19	39.6
G3 (Ki67 > 20%)	0	0
Unknown	6	12.5
Liver Metastases		
Yes	29	60.4
No	19	39.6
Liver Tumor Burden		
Type 0 (No Liver Metastases)	11	22.9
Type 1 (<5 Metastases Confined in 1 lobe)	12	25
Type 2 (Bilobar and/or 5–10 Metastases)	2	4.2
Type 3 (>10 Metastases or Diffuse Metastatic Disease)	15	31.3
Extrahepatic Metastases (Distant)		
Yes	6	12.5
No	42	87.5
Peritoneal Carcinomatosis		5
Yes	8	16.7
No	40	83.3
Staging		
Stage I	3	2.1
Stage II	11	6.3
Stage III	33	22.9
Stage IV		68.8
Prior Surgery		
Yes	43	89.6
No	5	10.4
Concomitant SSA Treatment		
Yes	28	58.3
No	20	41.7
Other Systemic Treatments (IF-a, Chemotherapy. PRRT, MTT)		
Yes	11	22.9
No	37	77.1
Charlson Comorbidity Index		
0	15	31.3
1	7	14.6
2	4	8.3
3	5	10.4
≥4	10	20.8
Serum 5HIAA levels, nmol/L		
≤123	20	41.7
123–250	12	25
250–500	9	18.8
>500	7	14.6
Urinary 5HIAA levels, μmol/L/24 h		
≤50	12	25
50–200	9	18.8
200–500	1	2.1
>500	2	4.1
not available concurrently to serum sampling	24	50

Abbreviations. 5HIAA: 5-Hydroxyindoleacetic Acid; IF-a; MTT: Molecular Targeted Therapy; PRRT: Peptide Receptor Radionuclide Therapy; SSA: Somatostatin Analogs; TNM: tumor-nodes-metastases; WHO: World Health Organization.

**Table 2:** Patient- and tumor-related parameters in relation to serum 5HIAA values at baseline measurements.

Characteristics	s-5HIAA Value (Median with Range; nmol/L)	*p*-Value *
Gender		0.747
Male	144 (43–836)	
Female	156 (39–3199)	
Age, Years		0.709
Group1: 45–71 years	124 (39–3199)	
Group 2: 71–89 years	164 (43–836)	
Inheritance		0.909
Sporadic	160 (39–3199)	
Familial	99.5 (50–836)	
Primary Tumor Site		0.261
Small Intestine	164 (39–3199)	
Other (Pancreas, Lung, UPO)	124 (43–490)	
Secretory Status		0.711
Functioning	164 (60–3199)	
Non-Functioning	128 (39–836)	
WHO Grading		0.174
G1 (Ki67 < 3%)	119 (39–614)	
G2 (Ki67 3–20%)	164 (43–3199)	
Liver Metastases		0.045
Yes	223 (43–3199)	
No	72 (39–521)	
Liver Tumor Burden Classification		0.041
No Liver Metastases	72 (39–521)	
Type 1 and 2	175.5 (50–836)	
Type 3 (>10 Metastases or Diffuse Metastatic disease).	412 (43–3199)	
Extrahepatic Metastases (Distant)		0.599
Yes	232 (48–2076)	
No	134.5 (39–3199)	
Peritoneal Carcinomatosis		0.394
Yes	255.5 (50–836)	
No	126.5 (39–3199)	
TNM Staging		0.398
stage I-II	98 (62–251)	
stage III	113 (48–521)	
stage IV	223 (43–3199)	
Prior Surgery		0.193
Yes	144 39–3199)	
No	272 (128–614)	
Concomitant SSA Treatment		0.332
Yes	118 (39–2076)	
No	175 (48–3199)	
Other Systemic Treatments (IF-a, Chemotherapy. PRRT, MTT)		0.031
Yes	296 (50–3199)	
No	124 (39–2076)	
Charlson Comorbidity Index		0.888
0–2	160 (39–836)	
≥3	187 (8–3199)	

* Mann-Whitney or Kruskal-Wallis test, as appropriate. Statistical significant *p*-values are highlighted in bold. Abbreviations. 5HIAA: 5-Hydroxyindoleacetic Acid; IF-a: intereferon-alpha; MTT: Molecular Targeted Therapy; PRRT: Peptide Receptor Radionuclide Therapy; SSA: Somatostatin Analogs; TNM: tumor-nodes-metastases; WHO: World Health Organization.

## Data Availability

The data presented in this study are available on request from the corresponding author. The data are not publicly available due to privacy restrictions.
